# Data on isoaspartylation of neuronal ELAVL proteins

**DOI:** 10.1016/j.dib.2016.11.034

**Published:** 2016-11-17

**Authors:** Mario A. Pulido, Meleeneh Kazarian DerHartunian, Prerna Sehgal, Ite A. Laird-Offringa

**Affiliations:** a Departments of Surgery and of Biochemistry and Molecular Medicine, Norris Comprehensive Cancer Center, Keck School of Medicine, University of Southern California, Los Angeles, CA, USA

**Keywords:** Auto-antigens, Autoimmunity, ELAVL, Isoaspartylation, Neuronal proteins, Small cell lung cancer, SCLC

## Abstract

This article contains experimental data examining the propensity of neuronal ELAVL proteins to become isoaspartylated. The data are related to the article “Isoaspartylation appears to trigger small cell lung cancer-associated autoimmunity against neuronal protein ELAVL4” (M.A. Pulido, M.K. DerHartunian, Z. Qin, E.M. Chung, D.S. Kang, A.W. Woodham, J.A. Tsou, R. Klooster, O. Akbari, L. Wang, W.M. Kast, S.V. Liu, J.J.G.M. Verschuuren, D.W. Aswad, I.A. Laird-Offringa, 2016) [1], in which it was reported that the N-terminal region of recombinant human ELAVL4 protein, incubated under physiological conditions, acquires a type of highly immunogenic protein damage. Here, we present Western blot analysis data generated by using an affinity-purified polyclonal rabbit antibody (raised against an N-terminal ELAVL4 isoaspartyl-converted peptide) to probe recombinant protein fragments of the other three members of the ELAVL family: the highly homologous neuronal ELAVL2 (HuB) and ELAVL3 (HuC), and the much less homologous ubiquitously expressed ELAVL1 (HuR).

**Specifications Table**

**Table:** 

Subject area	*Biology*
More specific subject area	*Cancer immunology*
Type of data	*Raw*
How data was acquired	*Western blot obtained on Biorad Fluor-S*^*TM*^*MultiImager.*
Data format	*Raw images cropped to fit into the figure were used.*
Experimental factors	*Recombinant N-terminal ELAVL proteins incubated under isoaspartylation-inducing conditions for 0, 1, 3 or 7 days*
Experimental features	*Amino acid sequence alignment and Western blot analysis*
Data source location	*Los Angeles, CA, USA*
Data accessibility	*Data is presented in this article*

**Value of the data**

• The data encourages the investigation of isoaspartylation of other neuronal autoantigens.
• The data encourages the investigation of the oligomerization of autoantigens that might be prone to isoaspartylation and allows comparison to other antigens.
• The data supports the generation of additional anti-isoaspartyl antisera by investigators, including antisera to other neuronal SCLC auto-antigens.

## 1 Data

**Fig. 1:**
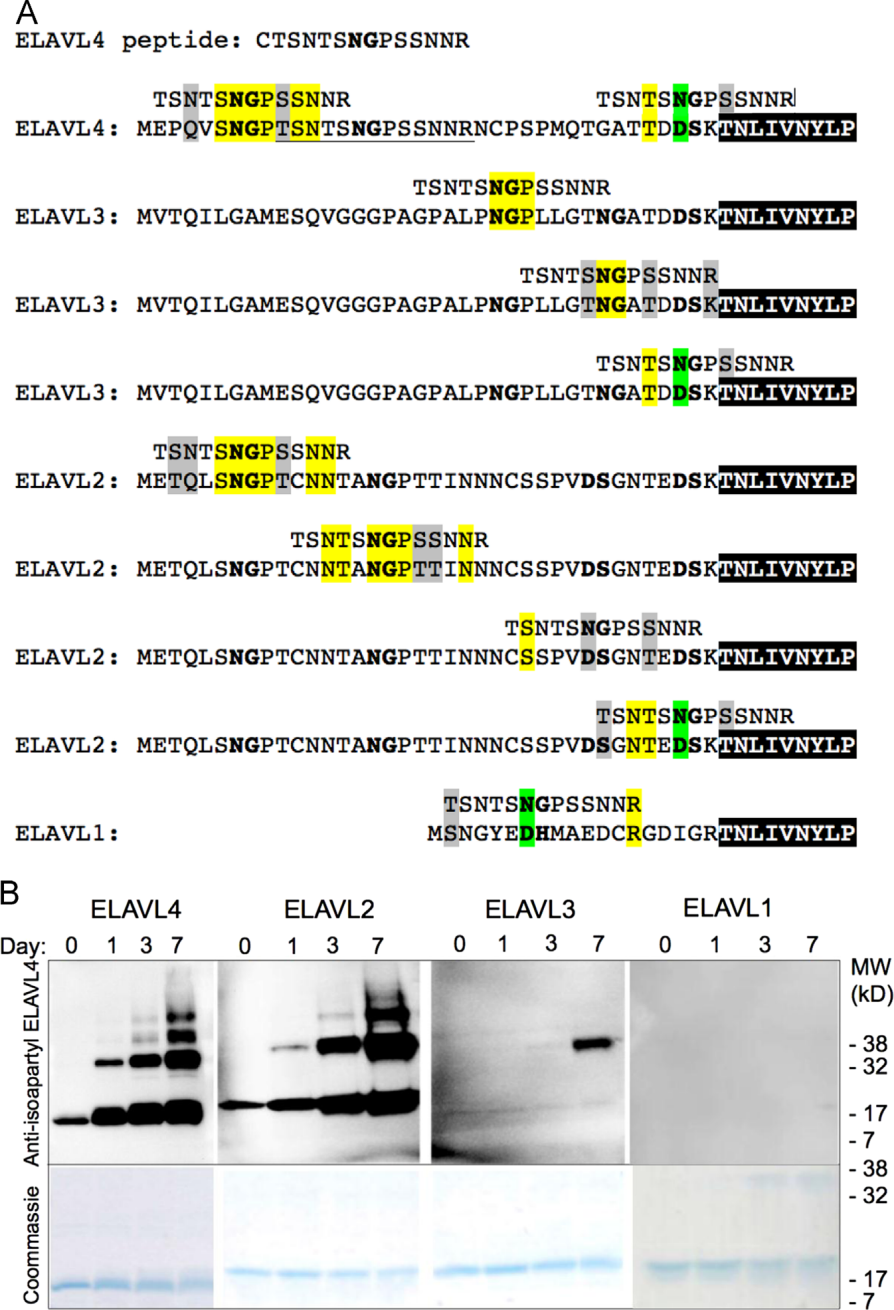
Alignment of ELAVL family members and examination of cross-reactivity with affinity-purified anti-isoaspartyl-ELAVL4 rabbit antiserum.

The data shown consists of two parts. First, we provide the alignment of the N-terminal amino acid sequences of ELAVL proteins, indicating regions of homology between the proteins and a peptide used to generate a rabbit anti-isoaspartyl-ELAVL4 serum [1]. Secondly, we show Western blots documenting the reactivity of the affinity-purified rabbit antiserum with recombinant human ELAVL2, ELAVL3, ELAVL4, and ELAVL1 polypeptides (consisting of the N-terminal parts of the proteins, including the first RNA recognition motif).

(A) Amino acid sequence alignments of neuronal ELAVL proteins (ELAVL2-4) and the ubiquitously-expressed ELAVL1 protein indicate any regions of homology with the peptide used to generate the rabbit anti-isoaspartyl-ELAVL4 antiserum. The peptide sequence is underlined, amino acids identical with the peptide sequence are highlighted in yellow and conserved substitutions are highlighted in gray, with N for D substitutions noted in green. Canonical isoaspartylation sites (N or D followed by S, G or H) are bolded. The N-terminal portion of the RNA recognition motif (RRM) domain is marked by the black box. ELAVL1 was included as a negative control because it has little homology to the peptide used to generate the anti-isoaspartyl-ELAVL4 serum.
(B) Western blot analysis of N-terminal regions of ELAVL proteins incubated under isoaspartyl-inducing conditions for 0, 1, 3 or 7 days (top panel). We have previously shown that the ELAVL4 aa 1–117 fragment, similar to the N-terminal fragment used to broadly characterize the ELAVL immune epitopes in numerous published studies (reviewed in [2]), becomes highly reactive with the affinity-purified anti-isoaspartyl-ELAVL4 rabbit antisera, and that both the monomer and oligomers that form during isoaspartyl conversion show reactivity to the sera [1]. Oligomerization is a known consequence of isoaspartylation [3], [4], [5], [6]. Just like ELAVL4, ELAVL2 and 3 acquire cross-reactivity with the anti-isoAsp-ELAVL4 antiserum during the isoaspartyl conversion (Fig. 1B, top panel). Reactivity with ELAVL2 closely resembles that of ELAVL4, while ELAVL3 only shows cross-reactivity in the dimer form. This may be because the homology between the N-terminal regions of ELAVL4 and ELAVL2 is higher than that of ELAVL3. The dimer proteins may exhibit alternative epitopes or show altered binding kinetics favoring antibody binding to the dimer, which may explain why reactivity with the ELAVL3 dimer is seen while reactivity with the monomer is not observed. As the Coomassie gel shows (Fig. 1B, bottom panel), the oligomers (which are resistant to denaturation) are present in modest amounts but appear to be unusually reactive. This figure is related to Figure 2 in Pulido et al. [1].

## 2 Experimental design, materials and methods

Recombinant human ELAVL2 (amino acids (aa) 1–117), ELAVL3 (aa 1–117), ELAVL4 (aa 1–117), and ELAVL1 (aa 1–98) polypeptides with C-terminal hexahistidine tags were produced in *E. coli* BL21(DE3) (New England Biolabs, Ipswich, MA, USA). The protein fragments contain the whole N-terminal RNA recognition motif (RRM) and amino acids N-terminal to it. Recombinant proteins were incubated under isoaspartyl-inducing conditions for 0, 1, 3 and 7 days (50 mM K-HEPES pH 7.4, 1 mM EGTA, 0.02% w/v sodium azide, 5% w/v glycerol at 37 °C as described [1]). 1 μg of each recombinant protein sample per lane was denatured for 10 min at 100 °C in reducing loading buffer (60 mM Tris–HCl pH 6.8, 2% SDS, 0.01% bromophenol blue, 5% 2-mercaptoethanol, 10% glycerol) and run on polyacrylamide gel electrophoresis-SDS gels (14%), transferred to Immun-Blot PVDF membranes (Bio-Rad, Hercules, CA, USA) and probed with an affinity-purified polyclonal rabbit antiserum [1] raised against CTSNTS-isoAsp-GPSSNNR-amide peptide carrying an isoaspartyl moiety at the central asparagine. The affinity-purified anti-isoaspartyl-ELAVL4 antiserum was used at 1:250 dilution in TBST (20 mM Tris/HCl, pH 8.0, 150 mM NaCl, 0.05% Tween 20) with 5% milk, washed in TBST and probed with a secondary goat anti-rabbit IgG HRP conjugate (at 1:20,000 in TBST with 3% milk) ( # sc-2004, Santa Cruz, CA, USA) followed by visualization with a chemiluminescent HRP substrate kit (Millipore, Danvers, MA, USA) on a Bio-Rad Fluor-STM MultiImager (BioRad, Hercules, CA, USA) according to the manufacturers’ instructions.

## References

[1] Pulido M.A., DerHartunian M.K., Qin Z., Chung E.M., Kang D.S., Woodham A.W., Tsou J.A., Klooster R., Akbari O., Wang L., Kast W.M., Liu S.V., Verschuuren J.J.G.M., Aswad D.W., Laird-Offringa I.A. (2016). Isoaspartylation appears to trigger small cell lung cancer-associated autoimmunity against neuronal protein ELAVL4. J. Neuroimmunol..

[2] Kazarian M., Laird-Offringa I.A. (2011). Small-cell lung cancer-associated autoantibodies: potential applications to cancer diagnosis, early detection, and therapy. Mol. Cancer.

[3] Kern R., Malki A., Abdallah J., Liebart J.C., Dubucs C., Yu M.H., Richarme G. (2005). Protein isoaspartate methyltransferase is a multicopy suppressor of protein aggregation in *Escherichia coli*. J. Bacteriol..

[4] Paranandi M.V., Aswad D.W. (1995). Spontaneous alterations in the covalent structure of synapsin I during in vitro aging. Biochem. Biophys. Res. Commun..

[5] Shimizu T., Watanabe A., Ogawara M., Mori H., Shirasawa T. (2000). Isoaspartate formation and neurodegeneration in Alzheimer׳s disease. Arch. Biochem. Biophys..

[6] Zirah S., Kozin S.A., Mazur A.K., Blond A., Cheminant M., Segalas-Milazzo I., Debey P., Rebuffat S. (2006). Structural changes of region 1-16 of the Alzheimer disease amyloid beta-peptide upon zinc binding and in vitro aging. J. Biol. Chem..

