# Supplementary material for: Data on isoaspartylation of neuronal ELAVL proteins
Source: Data Brief. 2016 Nov 17;9:1052–5. doi: 10.1016/j.dib.2016.11.034 (PMC5128017; doi:10.1016/j.dib.2016.11.034)
Supplement: Supplementary file 1 — Supplementary material [file mmc1.pdf]

AUTHOR DECLARATION

CONFLICT OF INTEREST FORM

We wish to draw the attention of the Editor to the following facts which may be considered as potential conflicts of interest and to significant financial contributions to this work.

Dr. Laird-Offringa, Dr. Pulido and Dr. Der Hartunian are co-inventors on a patent application: U.S. Provisional Application, No 61/697,165 Title: Methods and Compositions for Detecting, Imaging, and Treating Small Cell Lung Cancer Utilizing Post-Translationally Modified Residues and Higher Molecular Weight Antigenic Complexes in Proteins.

There are no other relationships/conditions/circumstances that present a potential conflict of interest

We confirm that the manuscript has been read and approved by all named authors and that there are no other persons who satisfied the criteria for authorship but are not listed. We further confirm that the order of authors listed in the manuscript has been approved by all of us.

We confirm that we have given due consideration to the protection of intellectual property associated with this work and that there are no impediments to publication, including the timing of publication, with respect to intellectual property. In so doing we confirm that we have followed the regulations of our institutions concerning intellectual property.

We understand that the Corresponding Author is the sole contact for the Editorial process (including Editorial Manager and direct communications with the office). He/she is responsible for communicating with the other authors about progress, submissions of revisions and final approval of proofs. We confirm that we have provided a current, correct email address which is accessible by the Corresponding Author and which has been configured to accept email from ilaird@usc.edu.

Signed by all authors as follows:

Mario A. Pulido — October 6, 2016

Mario A. Pulido — Date

6 October 2016

Meleeneh Kazarian DerHartunian — Date

Prerna Sehgal — October 6, 2016

Prerna Sehgal — Date

10/6/2016

Ite A. Laird-Offringa (corresponding author) — Date
